# Calorie Restricted High Protein Diets Downregulate Lipogenesis and Lower Intrahepatic Triglyceride Concentrations in Male Rats

**DOI:** 10.3390/nu8090571

**Published:** 2016-09-15

**Authors:** Lee M. Margolis, Donato A. Rivas, Yassine Ezzyat, Erin Gaffney-Stomberg, Andrew J. Young, James P. McClung, Roger A. Fielding, Stefan M. Pasiakos

**Affiliations:** 1 Nutrition, Exercise, Physiology, and Sarcopenia Laboratory, U.S. Department of Agriculture Jean Mayer Human Nutrition Research Center on Aging, Tufts University, Boston, MA 02111, USA; Lee.Margolis@Tufts.edu (L.M.M.); Donato.Rivas@Tufts.edu (D.A.R.); Yassine.Ezzyat@Tufts.edu (Y.E.); Roger.Fielding@Tufts.edu (R.A.F.); 2 Military Nutrition Division, U.S. Army Research Institute of Environmental Medicine, Natick, MA 01760, USA; Andrew.J.Young.ctr@mail.mil (A.J.Y.); James.P.McClung8.civ@mail.mil (J.P.M.); 3 Military Performance Division, U.S. Army Research Institute of Environmental Medicine, Natick, MA 01760, USA; Erin.G.Stomberg.civ@mail.mil

**Keywords:** low carbohydrate diet, fatty acid synthase, insulin resistance, fatty liver

## Abstract

The purpose of this investigation was to assess the influence of calorie restriction (CR) alone, higher-protein/lower-carbohydrate intake alone, and combined CR higher-protein/lower-carbohydrate intake on glucose homeostasis, hepatic de novo lipogenesis (DNL), and intrahepatic triglycerides. Twelve-week old male Sprague Dawley rats consumed ad libitum (AL) or CR (40% restriction), adequate (10%), or high (32%) protein (PRO) milk-based diets for 16 weeks. Metabolic profiles were assessed in serum, and intrahepatic triglyceride concentrations and molecular markers of de novo lipogenesis were determined in liver. Independent of calorie intake, 32% PRO tended to result in lower homeostatic model assessment of insulin resistance (HOMA-IR) values compared to 10% PRO, while insulin and homeostatic model assessment of β-cell function (HOMA-β) values were lower in CR than AL, regardless of protein intake. Intrahepatic triglyceride concentrations were 27.4 ± 4.5 and 11.7 ± 4.5 µmol·g^−1^ lower (*p* < 0.05) in CR and 32% PRO compared to AL and 10% PRO, respectively. Gene expression of fatty acid synthase (FASN), stearoyl-CoA destaurase-1 (SCD1) and pyruvate dehydrogenase kinase, isozyme 4 (PDK4) were 45% ± 1%, 23% ± 1%, and 57% ± 1% lower (*p* < 0.05), respectively, in CR than AL, regardless of protein intake. Total protein of FASN and SCD were 50% ± 1% and 26% ± 1% lower (*p* < 0.05) in 32% PRO compared to 10% PRO, independent of calorie intake. Results from this investigation provide evidence that the metabolic health benefits associated with CR—specifically reduction in intrahepatic triglyceride content—may be enhanced by consuming a higher-protein/lower-carbohydrate diet.

## 1. Introduction

Hepatic de novo lipogenesis (DNL) is the biochemical synthesis of fatty acids from acetyl-CoA subunits that result from glycolysis and carbohydrate metabolism. Consuming a high-carbohydrate diet can elevate circulating glucose and insulin concentrations, which upregulates DNL and intrahepatic fat accumulation [1]. This process occurs when excessive acetyl-CoA produced in the Kreb’s cycle is converted to malonyl CoA through reactions catalyzed by acetyl-CoA carboxylase (ACC), fatty acid synthase (FASN) and stearoyl-CoA desaturase (SCD). Excess fat accumulation in the liver may lead to (or exacerbate existing) peripheral insulin resistance and hypertriglyceridemia [2,3]. These metabolic dysfunctions are associated with the accumulation of total body fat, and, if left untreated, can result in nonalcoholic fatty liver disease and type 2 diabetes [4,5].

Reducing dietary energy intake and manipulating the macronutrient composition of the diet can prevent the development of and mitigate existing metabolic dysfunction. Caloric restriction (CR) lowers intrahepatic triglyceride content and enhances insulin sensitivity by reducing total body fat mass [6]. Increasing protein intake within the acceptable macronutrient distribution range (>10% but ≤35% of total energy intake) at the expense of carbohydrate (higher-protein/lower-carbohydrate diet) may further improve metabolic health with CR [7]. Higher-protein/lower-carbohydrate CR diets reduce circulating triglyceride concentrations and appear to stabilize postabsorptive glucose homeostasis [1,8] and reduce postprandial glycaemia and insulinemia [9,10,11]. These glycemic responses limit substrate availability for DNL, reduce intrahepatic triglyceride accumulation, and enhance insulin sensitivity. Although combining a higher-protein/lower-carbohydrate diet with CR appears to be beneficial, the independent versus synergistic effects of such dietary manipulations on glycemic control, DNL, and intrahepatic triglyceride concentrations are not well described.

The objectives of the present investigation were to assess the independent effects of CR, higher-protein/lower-carbohydrate intake, and the combined effects of both on glucose homeostasis, DNL, and intrahepatic triglyceride concentration after a 16 week controlled feeding intervention. We hypothesized that consuming a higher-protein/lower-carbohydrate diet during CR would reduce circulating glucose and insulin concentrations to a greater extent than CR alone and higher-protein ad libitum (AL) feeding. Additionally, we hypothesized that higher-protein diets alone would downregulate molecular regulators of DNL, and that combining CR with higher-protein feeding would confer the greatest glycemic and lipogenic advantage over either of the interventions alone.

## 2. Materials and Methods

### 2.1. Experimental Design

Twelve-week-old male Sprague Dawley rats (*n* = 40; Charles River Laboratories) were housed in a temperature-controlled room on a 12 h light–dark cycle. Following a two week acclimation phase, rats were randomly assigned to one of four diet groups consuming ad libitum (AL) or calorie restricted (CR; 40%) diets, with standard (10%) or high (32%) protein (PRO) content for 16 weeks. A total of 10 rats were randomized into each diet group. Only tissue samples from rats consuming milk-based protein diets were selected for this study, to eliminate protein source as a confounding variable, given that the intent of this study was to determine the independent and combined effects of altered protein and carbohydrate intake with AL or CR feeding. All study procedures were approved by the US Army Research Institute of Environmental Medicine Animal Care and Use Committee. This prolonged study in healthy male rats was part of a larger investigation [12] assessing the influence of energy status and dietary protein level and source on bone metabolism. Non-obese, healthy male rats were studied to reflect the population of military service members that are most likely to be exposed to prolonged underfeeding during extended military operations. Identifying ways to improve metabolic health in this population by manipulating the macronutrient composition of a calorie restricted diet is a primary focus of our research program.

### 2.2. Dietary Intervention

Purified study diets based on AIN-93 (Dyets, Inc., Bethlehem, PA, USA) were modified to provide 10% and 32% protein to reflect the lower and upper end of the current acceptable macronutrient distribution range (milk protein concentrate, Idaho Milk Products, Jerome, ID, USA) [12]. Chemical analysis of diets was performed to ensure nutrient content (Covance Laboratories, Dedham, MA, USA). The amount of feed provided to the CR rats was initially determined by averaging daily intake (26 g·day^−1^) during the 14 day acclimation phase. Feed intake for CR fed rats was 16 ± 2 g·day^−1^, whereas the AL rats consumed 26 ± 3 g·day^−1^. Feed intake for the AL rats was assessed every 2 days, such that adjustments could be made to ensure the CR rats maintained a 40% energy deficit. Complete dietary intake data has been reported [12]. Macronutrient content (% energy) of the diets was 10% protein, 20% fat, and 70% carbohydrate (Adequate); and 32% protein, 22% fat, and 46% carbohydrate (PRO). The carbohydrate-to-protein ratio for rats consuming the 10% PRO diet was 7:1 g·day^−1^, whereas the ratio for rats consuming the 32% PRO diet was 1.3:1 g·day^−1^.

### 2.3. Total Body and Fat Mass

Dual Energy X-ray Absorptiometry (DXA; Lunar iDXA, GE Lunar Corp., Madison, WI, USA) was utilized to assess total body and fat mass (FM). Rats were anesthetized by intraperitoneal injection of a 1 mL·kg^−1^ mixture of 40 mg·kg^−1^ ketamine (Ketaset; Fort Dodge Animal Health, Fort Dodge, IA, USA), 10 mg·kg^−1^ xylazine (Xyla-Ject; Phoenix Scientific, Inc., St. Joseph, MO, USA), and 1.0 mg·kg^−1^ acepromazine (Boehringer Ingelheim, St. Joseph, MO, USA) to ensure they remained still during the DXA scan. Small animal software (enCore Version 11.40.004, 2007; GE Lunar Corp, Madison, WI, USA) was utilized for the determination of body composition.

### 2.4. Metabolic Profile

Fasting blood was collected by cardiac puncture at the conclusion of the 16 week feeding intervention. Glucose, insulin, glucagon, and leptin concentrations were measured in serum using a MILLIPLEX^®^ MAP Rat Metabolic Magnetic Bead Panel Kit (Millipore, Billerica, MA, USA) on a Luminex 100 instrument (Luminex Co., Austin, TX, USA) using the appropriate software (Bio-Plex Version 5.0, Bio-Rad, Hercules, CA, USA). Serum triglyceride concentrations were determined using a commercially available colorimetric assay (Sigma, St. Louise, MO, USA) on an ELx808 Absorbance Reader (BioTek^®^, Winooski, VT, USA).

Homeostatic model assessment of insulin resistance (HOMA-IR) and β-cell function (HOMA-β) were calculated as estimates of glycemic homeostasis, where:

HOMA-IR = Glucose (mmol·L^−1^) × Insulin (µU·mL^−1^)/22.5 (1)

HOMA-β = 20 × Insulin (µU·mL^−1^))/Glucose (mmol·L^−1^) − 3.5 (2)

### 2.5. Hepatic Triglyceride Concentrations

Intrahepatic triglyceride concentrations were determined using the Folch method [13]. In brief, 20 mg (wet weight) of liver tissue was homogenized in a 2:1 chloroform-to-methanol solvent. Following extraction, samples were saponified in ethanolic KOH at 60 °C, and glycerol content was determined using a commercially available colorimetric assay (Sigma, St. Louise, MO, USA) on an ELx808 Absorbance Reader (BioTek^®^, Winooski, VT, USA).

### 2.6. mRNA Expression

Changes in mRNA expression for genes associated with lipogenesis (acetyl-CoA carboxylase, ACC; fatty acid synthase, FASN; stearoyl-CoA desaturase, SCD1; and pyruvate dehydrogenase kinase, isozyme 4, PDK4) were determined using commercially available primers (Qiagen, Waltham, MA, USA). Total RNA was isolated from 20 mg of liver tissue using Aurum™ Total RNA Fatty and Fibrous Tissue Kit (Bio-Rad, Hercules, CA, USA). Quantity and quality of isolated RNA were assessed using a Nanodrop ND-1000 spectrophotometer (Nanodrop, Wilmington, DE, USA). Equal amounts of total RNA (250 ng) were synthesized into cDNA using iScript™ Advanced cDNA Synthesis Kit (Bio-Rad). Reverse transcription was conducted using a T100™ Thermal Cycler (Bio-Rad). Samples were run in 20 μL reactions in triplicate, using iTaq™ Universal SYBR^®^ Green Supermix (Bio-Rad) for RT-qPCR amplifications performed on a CFX96 Touch™ Real-Time PCR Detection System (Bio-Rad). All target mRNA were normalized to the ribosomal protein L32 mRNA as an endogenous control. Fold changes for mRNA were calculated using the ∆∆ cycle threshold (∆∆C*_T_*) method [14], with fold changes expressed relative to the mean values for the control group, AL 10% PRO.

### 2.7. Protein Expression

Western blotting was performed to quantify the total protein content of molecular markers associated with the regulation of lipogenesis. Liver tissue (~30 mg) was homogenized on ice-cold homogenization buffer (1:10 *w/v*) containing 50 mM Tris–HCl (pH 7.5), 5 mM Na-pyrophosphate, 50 mM NaF, 1 mM EDTA, 1 mM Ethylene glycol tetraacetic acid (EGTA), 10% glycerol (*v/v*), 1% Triton-X, 1 mM Dithiothreitol (DTT), 1 mM benz-amidine, 1 mM phenylmethane sulfonyl fluoride (PMSF), 10 μg·mL^−1^ trypsin inhibitor, and 2 μg·mL^−1^ aprotinin. Following homogenization, samples were centrifuged for 15 min at 10,000× *g* at 4 °C, the supernatant (lysate) was collected, and protein content was determined using the 660 nm Protein Assay (ThermoFisher Scientific, Waltham, MA, USA).

Tissue lysates were solubilized in Laemmli buffer with equal amounts of total protein (10 µg) and separated by SDS-PAGE using precast Tris·HCl gels (Bio-Rad). Protein was transferred to polyvinylidene fluoride membranes and exposed to commercially available primary antibodies specific to ACC, FASN (Cell Signaling Technology, Danvers, MA, USA), and SCD (Abcam, Cambridge, MA, USA) at 4 °C overnight. Labeling was performed using a secondary antibody (anti-rabbit IgG conjugate with horseradish peroxidase; Cell Signaling Technology, Danvers, MA, USA), and chemiluminescent reagent was applied (Super Signal, West Pico Kit; Pierce Biotechnology, Rockford, IL, USA). Phosphoimager (ChemiDoc XRS; Bio-Rad), and Image Lab software (Bio-Rad) were used to quantify Western blot band density. Total protein data were normalized to Heat Shock Protein 90 (HSP90) to confirm equal protein loading. All data are presented as fold change compared to AL 10% PRO.

### 2.8. Statistical Analysis

Univariate ANOVA was conducted to assess the effects of calorie (AL vs. CR) and protein intake (10% PRO vs. 32% PRO) on body and fat mass, blood analytes, glucose homeostasis, hepatic triglyceride concentrations, Western blots, and gene expression. If a significant interaction (energy-by-time) was detected, Bonferroni adjustments for multiple comparisons were performed. Spearman’s rho correlation coefficients were used to determine the relationship between FASN and PDK4 gene expression, whole-body fat mass, insulin concentrations, and intrahepatic triglyceride concentrations. Gene expression data reported as fold regulation for correlations to maintain equal scale between positive and negative numbers. All data are presented as mean ± SEM. The α level for significances was *p* < 0.05. Data were analyzed using IBM SPSS Statistics for Windows Version 22.0 (IBM Corp., Armonk, NY, USA).

## 3. Results

### 3.1. Total Body and Fat Mass

At the conclusion of the 16 week feeding study, body mass in AL-fed rats (AL 10% PRO 550 ± 14 g, AL 32% PRO 550 ± 15 g) was higher (*p* < 0.05, calorie main effect) than CR-fed rats (CR 10% PRO 448 ± 5 g, CR 32% PRO 447 ± 10 g). Similarly, fat mass in AL fed rats (AL 10% PRO 227 ± 6 g, AL 32% PRO 222 ± 15 g) was greater (*p* < 0.05, calorie main effect) than CR fed rats (CR 10% PRO, 135 ± 9 g; CR 32% PRO 131 ± 7 g) after the 16 week intervention. No effect of dietary protein intake was observed on total body or fat mass.

### 3.2. Metabolic Profile

Main effects of calorie and protein were observed for leptin, as concentrations for CR and 32% PRO being lower (*p* < 0.05) than AL and 10% PRO, respectively (Table 1). Regardless of dietary protein intake, a main effect of calorie was observed for serum triglyceride and insulin concentrations, as well as HOMA-β, with values for CR being lower (*p* < 0.05) compared to AL (Table 1).

No effects of calories or protein were observed for glucose concentrations and HOMA-IR (Table 1). However, when statistical outliers (>2 standard deviations from the mean) were removed, a main effect of protein (*p* < 0.05) was observed for glucose concentration (AL 10% PRO: 8.0 ± 0.9, AL 32% PRO: 6.2 ± 0.3, CR 10% PRO: 8.3 ± 1.0, CR 32% PRO: 5.9 ± 0.1 mmol·L^−1^) and HOMA-IR (AL 10% PRO: 9.6 ± 1.4, AL 32% PRO: 6.8 ± 1.3, CR 10% PRO: 8.4 ± 2.0, CR 32% PRO: 5.0 ± 0.7), regardless of calorie intake.

### 3.3. Hepatic Triglyceride Concentration

**Figure 1:**
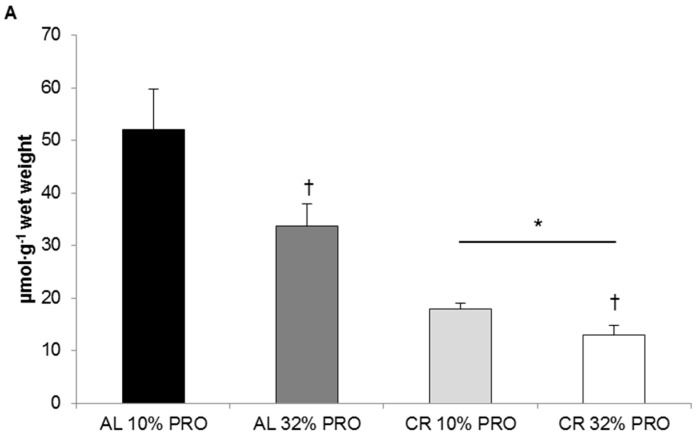
Intrahepatic triglyceride content. Values presented as mean ± SEM. * Main effect of energy status, calorie restriction (CR) different than ad libitum (AL); *p* < 0.05. ^†^ Main effect of protein (PRO), 32% PRO different than 10% PRO; *p* < 0.05.

Intrahepatic triglyceride concentrations for CR were 27.4 ± 4.5 µmol·g^−1^ lower (*p* < 0.05, calorie main effect) than AL (Figure 1). Similarly, intrahepatic triglycerides for 32% PRO were 11.7 ± 4.5 µmol·g^−1^ lower (*p* < 0.05, protein main effect) than 10% PRO.

### 3.4. mRNA Expression

**Figure 2:**
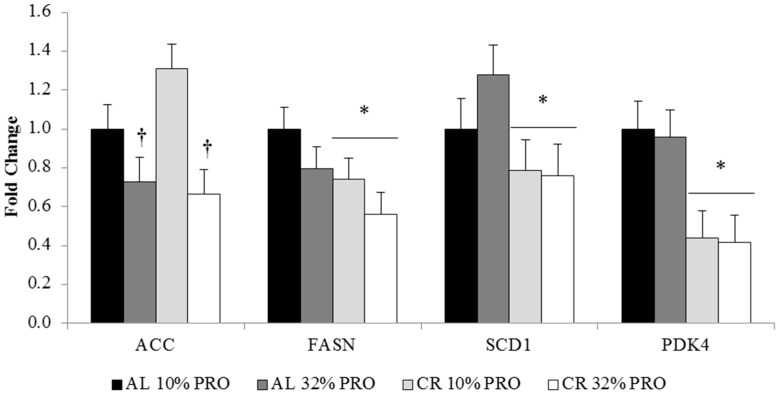
mRNA expression of lipogenic regulators. Values presented as mean ± SEM. * Main effect of calorie intake, CR different than AL; *p* < 0.05. ^†^ Main effect of protein intake, 32% PRO different than 10% PRO; *p* < 0.05. ACC: acetyl-CoA carboxylase; FASN: fatty acid synthase; SCD1: stearoyl-CoA desaturase; PDK4: pyruvate dehydrogenase kinase, isozyme 4.

mRNA expression of ACC was downregulated (*p* < 0.05) 30% ± 1% fold with 32% PRO versus 10% PRO, independent of calorie intake (Figure 2). FASN, SCD1, and PDK4 expression were (*p* < 0.05) 45% ± 1%, 23% ± 1%, and 57% ± 1% lower, respectively, in CR compared to AL, regardless of protein intake.

### 3.5. Protein Expression

**Figure 3:**
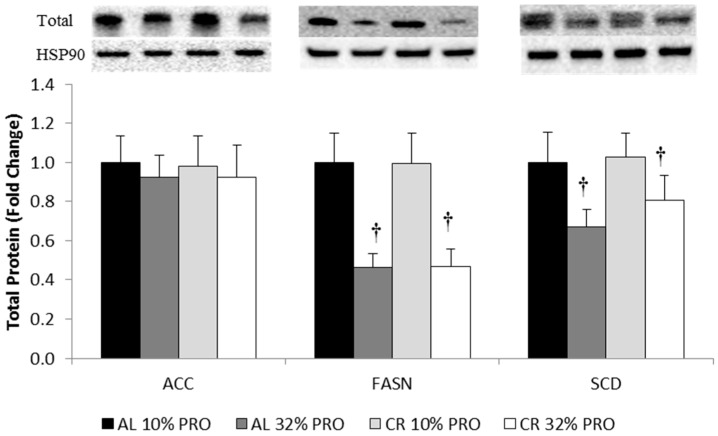
Total protein expression of lipogenic regulators relative to AL 10% PRO, normalized to HSP90. Values presented as mean ± SEM. ^†^ Main effect of protein, 32% PRO different than 10% PRO; *p* < 0.05. HSP90: heat shock protein 90.

Total protein for FASN was 50% ± 1% lower (*p* < 0.05) in 32% PRO compared to 10% PRO (Figure 3). Similarly, 32% PRO resulted in SCD total protein being 26% ± 1% lower (*p* < 0.05) compared to 10% PRO. There was no effect of calorie intake for total protein expression of FASN and SCD. No effect of calorie or protein intake was observed in total protein expression of ACC.

### 3.6. Relationship of Whole-Body Fat, Intrahepatic Triglycerides, and Gene Expression

**Figure 4:**
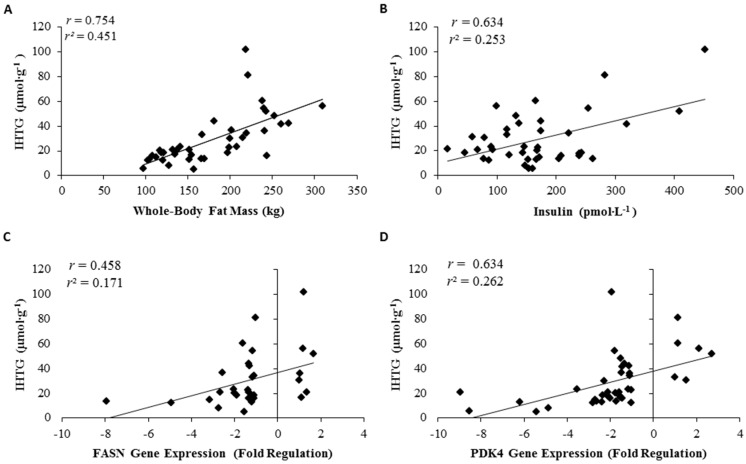
Correlation of intrahepatic triglycerides (IHTG) to whole-body fat (**A**); IHTG to serum insulin (**B**); IHTG to FASN gene expression (**C**); and IHTG to PDK4 gene expression (**D**). All correlations significant; *p* < 0.05.

Whole-body fat mass was positively associated with intrahepatic triglyceride concentrations (*r* = 0.754, *r^2^* = 0.451; *p* < 0.01; Figure 4A). Intrahepatic triglycerides were positively associated with circulating insulin concentrations (*r* = 0.634, *r^2^* = 0.253; *p* < 0.01; Figure 4B) and FASN gene expression (*r* = 0.458, *r^2^* = 0.171; *p* < 0.01; Figure 4C). Additionally, a positive correlation was observed with PDK4 and intrahepatic triglycerides (*r* = 0.634, *r^2^* = 0.262; *p* < 0.01; Figure 4D).

## 4. Discussion

The primary findings from this study were that reducing the ratio of dietary carbohydrate-to-protein (7:1 vs. 1.3:1) lowered intrahepatic triglycerides, particularly when combined with CR. The hepatic health benefits of consuming a higher-protein CR diet appeared to be driven by the downregulation of DNL caused by reduced gene expression and total protein content of several molecular regulators of intrahepatic lipogenesis. Reducing the dietary carbohydrate-to-protein ratio also tended to improve glycemic control, as indicated by HOMA-IR (e.g., estimate of insulin sensitivity), while CR independently improved HOMA-β (e.g., estimate of pancreatic β-cell function). These findings confirm the independent metabolic advantages of dietary energy and protein manipulations, as well as demonstrate that benefits are more pronounced when CR and higher-protein/lower-carbohydrate diets are combined.

Excess body fat—particularly visceral fat—is often accompanied by the accumulation of intrahepatic triglycerides [15]. An increase in intrahepatic triglyceride concentrations can inhibit insulin receptor kinase activity, leading to impaired glucose homeostasis and insulin resistance [16,17,18]. Findings from the present investigation, supported by others [19,20], indicate that nutrition interventions can target multiple metabolic dysfunctions, as total body fat mass, circulating insulin concentrations, HOMA-β, and intrahepatic triglyceride concentrations were lower in CR compared to AL fed rats. Though not statistically significant, rats fed higher-protein (32% PRO) tended to improve glucose and HOMA-IR compared to standard protein (10% kcal), regardless of energy intake. Interestingly, several investigations have suggested that increased protein intake may be detrimental to glucose homeostasis [21,22] because elevated branched-chain amino acid (BCAA) concentrations have been associated with insulin resistance [23,24]. Some suggest that amino acid-induced activation of the mechanistic target of rapamycin complex 1 (mTORC1) inhibits activity of insulin receptor substrate 1, disrupting insulin signaling and thus impairing glucose homeostasis [25]. However, findings from the present and past studies [8,9,11,26,27] have shown that consuming a higher-protein/lower-carbohydrate diet stabilizes or at least maintains glycemic control. Varying results between investigations may be attributed to methodological discrepancies. Several investigations reporting negative associations between insulin sensitivity and elevated BCAA concentrations isolated signal nutrients by employing BCAA-deficient diets to show an effect [28,29]. In the present study and others reporting a benefit of increased protein intake [8,9,11,26,27], investigators manipulated multiple macronutrients, with elevated protein intake matched by reductions in carbohydrate intake. Likely, the combination of alterations in protein and carbohydrate intake causes lower postprandial glycemic and insulinemic responses to meals, upregulated postabsorptive gluconeogenesis, and thus enhanced glycemic control over time [7].

Along with alterations in circulating insulin concentrations and intrahepatic triglycerides, a downregulation in the gene expression of PDK4 was observed with CR. PDK4 is an inhibitor of pyruvate dehydrogenase, which is the enzyme required for the entry of metabolites derived from glycolysis into the Kreb’s cycle [30]. The expression of PDK4 is sensitive to insulin, as insulin resistance results in an upregulation of PDK4 expression [31]. Following reductions in triglyceride concentrations at the tissue level with prolonged starvation, declines in the expression PDK4 have been shown to be the result of greater insulin sensitivity, allowing for improved glucose uptake and metabolism [32,33]. Diminished PDK4 gene expression with CR and correlations to intrahepatic triglyceride concentrations in the current study further highlights the relationship between excessive lipid accumulation in the liver and glucose metabolism.

Lower intrahepatic triglyceride concentrations in the present investigation may be the result of downregulated DNL. The synthesis of endogenous fatty acids is due to over-consumption of calories and dietary carbohydrate, resulting in an upregulation in ACC, FASN, and SCD activity [34,35,36]. As such, CR and lower-carbohydrate diets diminish DNL and intrahepatic triglycerides [37,38,39,40]. The present study is the first to examine their independent and synergistic effects on molecular regulators of DNL, particularly in non-obese, healthy rats, even though previous investigations have reported benefits of CR and altered macronutrient intake. Interestingly, the independent effects of altered calorie and carbohydrate-to-protein intake on molecular regulators of DNL appear to target distinct regulatory processes. Independent of macronutrient intake, CR primarily drove the downregulation of FASN and SCD1 gene expression (e.g., transcription), while higher-protein/lower-carbohydrate intake diminished total hepatic protein content of FASN and SCD (e.g., translation) regardless of calorie intake. While no calorie-by-protein interactions were observed on gene or protein expression, the independent effects of CR and higher-protein/lower-carbohydrate intake appear to culminate into a synergistic benefit, as rats fed CR 32% PRO diets had the lowest levels of intrahepatic triglyceride concentrations.

## 5. Conclusions

In conclusion, this study demonstrated that CR and higher-protein/lower-carbohydrate diets independently downregulate the transcription and translation of molecular markers regulating DNL. Furthermore, these two dietary interventions elicit an apparent synergistic benefit by reducing intrahepatic triglyceride concentrations. These data suggest that combining restricted carbohydrate and elevated dietary protein intake during periods of CR may target multiple metabolic processes that may improve long-term health.

## Figures and Tables

**Table 1:** Circulating Metabolic Profile ^1^.

Analytes	Ad Libitum		Calorie Restriction		*p* Value		
	10% PRO	32% PRO	10% PRO	32% PRO	Calorie	Protein	C × P ^2^
Glucagon (ng·L^−1^)	50.7 ± 4.2	55.3 ± 5.4	49.8 ± 3.2	50.3 ± 3.3	0.48	0.55	0.62
Leptin (mmol·L^−1^)	3912.4 ± 336.7	3019.4 ± 327.8	2035.0 ± 184.7	1756.3 ± 184.7	<0.01 *	0.03 ^†^	0.26
Triglycerides (mmol·L^−1^)	0.95 ± 0.12	0.83 ± 0.11	0.69 ± 0.03	0.57 ± 0.07	<0.01 *	0.22	0.98
Glucose (mmol·L^−1^)	8.0 ± 0.9	7.0 ± 0.8	8.3 ± 1.0	7.0 ± 0.8	0.83	0.19	0.90
Insulin (pmol·L^−1^)	234.1 ± 37.7	161.7 ± 26.9	144.3 ± 24.5	137.6 ± 24.5	0.04 *	0.15	0.23
HOMA-IR ^3^	12.7 ± 3.3	7.2 ± 1.2	8.4 ± 2.0	6.3 ± 1.0	0.22	0.08	0.40
HOMA-*B* ^4^	58.5 ± 9.0	49.3 ± 8.1	35.4 ± 5.5	41.4 ± 5.5	0.04 *	0.83	0.30

^1^ Values mean ± SEM; ^2^ C × P: Calorie-by-Protein interaction; ^3^ Homeostatic model assessment of insulin resistance; ^4^ Homeostatic model assessment of β-cell function * Calorie Restriction lower than Ad Libitum feeding; *p* < 0.05. ^†^ 32% PRO lower than 10% PRO; *p* < 0.05.

## Abbreviations

The following abbreviations are used in this manuscript:

ACC	Acetyl CoA Carboxylase
AL	Ad libitum
BCAA	Branched chain amino acids
CR	Calorie restriction
DNL	de novo lipogenesis
DXA	Dual Energy X-ray Absorptiometry
FASN	Fatty acid synthase
FM	Fat mass
HOMA-IR	Homeostatic model assessment of insulin resistance
HOMA-β	Homeostatic model assessment of β-cell function
HSP90	Heat shock protein 90
IHTG	Intrahepatic triglycerides
mTORC1	mechanistic target of rapamycin complex 1
PDK4	Pyruvate dehydrogenase kinase, isozyme 4
PRO	Protein
SCD1	Stearoyl-CoA destaurase
